# Toward Natural Wound Healing Therapy: Honey and *Calendula officinalis* Loaded κ-Carrageenan Films with Promising Hemostatic Potential

**DOI:** 10.3390/pharmaceutics17050578

**Published:** 2025-04-28

**Authors:** Jovana S. Vuković, Srđan Perišić, Anja Nikolić, Ivan Milošević, Milorad Mirilović, Bogomir Bolka Prokić, Tijana Lužajić Božinovski

**Affiliations:** 1 Innovation Center of the Faculty of Technology and Metallurgy, University of Belgrade, Karnegijeva 4, 11000 Belgrade, Serbia; 2 Faculty of Veterinary Medicine, University of Belgrade, Bulevar Oslobođenja 18, 11000 Belgrade, Serbia

**Keywords:** κ-carrageenan, honey, *Calendula officinalis*, hemostasis, wound dressing, wound healing, hemostatic potential

## Abstract

**Background/Objectives**: Efficient wound treatment embraces the management of four overlapping phases, starting with hemostasis, an immediate physiological response aimed at stopping bleeding from damaged blood vessels caused by skin injury. This paper proposes an innovative, nature-based hemostatic biomaterial designed to assist natural self-healing regenerative mechanisms. **Methods**: Light, transparent, and skin-adhesive films based on κ-carrageenan, meadow polyfloral honey, and *Calendula officinalis* flower extract were fabricated via solution casting. Comprehensive characterization revealed the physicochemical, structural, swelling, and barrier properties and the influence of each bioactive compound utilized for film preparation. **Results**: The samples subcutaneously implanted in Wistar rats induced vascularization, deposition of collagen, and orientation of collagen fibers while being fully phagocytosed and gradually biodegraded. The rat tail-cut model demonstrated that the films significantly reduced blood loss (0.1875 ± 0.0732 g) compared to the control (0.7837 ± 0.3319 g), and hemostasis was achieved notably faster (355.75 ± 71.42 s) than in the control group (704.25 ± 85.29 s). The rat liver punch biopsy model confirmed reduced blood loss (2.8025 ± 1.5174 g) and shorter time to hemostasis (303.25 ± 77.90 s) compared to the control (3.1475 ± 1.5413 g, 383.00 ± 36.53 s). **Conclusions**: The results indicate the great potential of the fabricated films as hemostatic wound dressings.

## 1. Introduction

A wound is a form of tissue disruption that affects its anatomical structure and physiological processes, thereby affecting the primal skin to protect the entire organism from harmful external factors [1]. The process of wound healing naturally undergoes a mechanism, which comprises four overlapping phases: hemostasis, inflammation, proliferation, and remodeling [2]. Hemostasis, the first phase of wound healing, is crucial because it aims to immediately stop bleeding from injured blood vessels caused by skin trauma. By properly managing hemostasis, the subsequent process of healing can take place unhindered by heavy blood loss and associated physiological complications. The rapid physiological response of the body to terminate bleeding involves vasoconstriction, platelet plug formation, and blood coagulation [3]. The mechanism of hemostasis starts with the secretion of vWF (von Willebrand factor) [4], followed by vascular contraction of the compromised blood vessel and reduction of blood flow. Collagen is activated, promoting platelets to adhere, aggregate, and finally form a plug. When a platelet plug is formed, the coagulation cascade begins with the activation of clotting factors and the translation of fibrinogen to fibrin. Fibrin produces a stabilizing mesh around the platelet plug, ensuring bleeding termination. The plug becomes more solid as the blood cells are caught in the mesh, which finalizes with the formation of a thrombus (clot) [5]. Efficient hemostasis provides normal continuation of the healing process through the next phases of inflammation, proliferation, and remodeling. The skin naturally possesses perfectly orchestrated self-healing mechanisms, which enable complete tissue regeneration in the case of minor, acute injuries. On the other hand, severely injured skin tissue and chronic full-thickness wounds involve microvascular damage, which increases the risk of complications, such as hemorrhage and infection, potentially resulting in prolonged or failed healing. Therefore, the development of advanced biomaterials for wound treatment has significantly increased in recent decades. A multidisciplinary approach to wound dressing development has a pivotal purpose: to support and promote each phase of the healing process, providing efficient regeneration of skin tissue.

Hemostatic wound dressings, along with suitable hemostatic activity, should possess the capacity to maintain an optimal moist environment for a wound to heal, the beneficial flow of gases and nutrients, proper adhesiveness, easy application and removal, protection of the wound from external harmful influences, prevention of infections, while expressing full biocompatibility with living cells [6,7]. Therefore, careful selection of the components is crucial, especially when the synergistic action of all components can lead to better results. In the pursuit of the most efficient and adequate wound treatment, scientists have recognized that the research strategy points in one direction: to mimic the nature and delicate processes within the body and assist them until regeneration is complete [8]. Biopolymer-based wound dressings provide inherent biocompatibility, bioactivity, biodegradability, and bioadhesivity compared to those originating from synthetic polymers [9]. Natural polysaccharides can construct hydrophilic, three-dimensional porous structures with biological and physicochemical properties that closely resemble those of the natural extracellular matrix (ECM) [10]. In recent years, polysaccharide-based hemostatic wound dressings have attracted attention due to their biocompatibility, biodegradability, low cost, natural and renewable resources, and variety of forms (hydrogel, film, gauze, sponge, and microsphere) [11,12].

Carrageenans are polysaccharides extracted from red seaweeds, consisting of long linear chains of D-galactose and D-anhydrogalactose with anionic sulfate groups. Based on the number of -SO_3_^−^ groups on the repeating disaccharide units, carrageenans are categorized as kappa (κ), iota (ι), and lambda (λ) carrageenans [13]. As one of the most abundant polysaccharides, κ-carrageenan possesses a great ability to form gels and resembles natural glycosaminoglycans while being a hydrophilic, biocompatible, and environmentally friendly biomacromolecule [14]. Numerous studies have revealed various inherent bioactive features of κ-carrageenan, such as antioxidant, antibacterial, and antiviral activities [15,16,17], which is why κ-carrageenan has been recognized as a promising candidate for tissue engineering and wound healing management [18,19,20,21]. κ-Carrageenan was employed as a component, along with carboxymethyl chitosan and polyvinyl alcohol, for the preparation of hemostatic membranes loaded with the antifibrinolytic drug tranexamic acid [22]. The electrospun κ-carrageenan exhibited better hemostatic activity than carboxymethyl chitosan, with in vivo assays showing the capacity of the fabricated membranes to stop bleeding for 38.4 s and 37.0 s in the liver and tail, respectively. Biranje et al. proposed a chitosan/carrageenan wound dressing for the treatment of traumatic hemorrhage [23]. The hemostatic potential of the freeze-dried samples was evaluated using blood clotting kinetics, adhesion of red blood cells and platelets, and thrombin generation assays. Chitosan/carrageenan dressings increased the adhesion and aggregation of blood cells and platelets, cell attachment, and improved hemostatic activity. Gelatin-tannic acid-κ-carrageenan (GTC) microparticles developed for rapid hemostasis-induced clot formation in 50 s and blood loss of 46 mg in the femoral artery of mice, compared to the control group with a clot formation time of 250 s and blood loss of 259 mg [24].

Honey, a natural compound produced by honeybees, is a viscous mixture composed of water, fructose, glucose, maltose, sucrose, enzymes, proteins, and amino acids [25]. Since ancient times, it has been known for its extraordinary bioactivity, especially in the treatment of different types of wounds, including chronic ones [26]. The unique composition of honey promotes each phase of wound healing by manifesting highly potent antioxidant, anti-inflammatory, antibacterial, and proangiogenic activities [27,28,29]. Honey and honey-based dressings significantly enhance collagen synthesis, angiogenesis, granulation, epithelization, and wound contraction while protecting the wound from bacterial infections [30,31]. Although some studies have revealed honey’s inhibitory effect on platelet aggregation and blood clotting [32], there is a significant influence on the inflammation phase via activation of anti-inflammatory mechanisms that suppress the production of pro-inflammatory cytokines and stimulate the production of inflammatory mediators prostaglandin E2 (PGE2) and cyclooxygenase-2 [33,34]. The inflammatory phase overlaps with hemostasis, and the actions are interlinked to ensure efficient wound healing. While hemostasis provides clot formation, the inflammatory phase maintains the stability of the formed clot by controlling infection and secretion of cytokines.

*Calendula officinalis* (*C. officinalis*) belongs to the *Asteraceae* family and is a traditional plant with wide medicinal use. The pharmacological significance of *C. officinalis* is attributed to its bioactive constituents, including triterpenoids, flavonoids, polyphenols, coumarins, quinones, oils, carotenoids, and amino acids [35,36]. These phytoconstituents initiate multifaceted therapeutic effects, such as anti-inflammatory [37], antioxidant [38,39], antimicrobial [40,41,42], and proliferative effects [43]. *C. officinalis* promotes wound healing by reducing inflammation, enhancing angiogenesis and protecting the wound from bacterial infections [44,45]. However, the application of *C. officinalis* in hemostatic wound dressing formulations has rarely been explored. Pal et al. exploited the hemostatic potential of *C. officinalis* extracts in butanol and water by performing a prothrombin time (PT) test [46]. The water extract induced faster coagulation (1 min) than the butanol extract (4 min) in contact with citrated plasma. Gelatin sponges loaded with *C. officinalis* were evaluated as hemostatic materials and blood clot formation was measured using the Lee–White method [47]. The presence of 7% *C. officinalis* oil in gelatin sponges reduced the time for clot formation from 161.70 ± 3.11 s to 158.75 ± 4.60 s, proving its hemostatic potential.

Here, we propose a novel natural medical device, wound dressings with hemostatic activity and synergistic therapeutic performance of carefully selected natural components for efficient wound treatment. A series of films based on κ-carrageenan, meadow polyfloral honey, and *C. officinalis* were successfully fabricated using the solution casting method. The concentrations of honey and *C. officinalis* were varied to determine the optimal composition of the films. Comprehensive characterization of the films was conducted, investigating their physico-chemical, structural, swelling, and barrier properties, and the influence of each bioactive component. The bioactivity and biocompatibility of the films were investigated in vivo by subcutaneous implantation in Wistar rats. The hemostatic effectiveness of the films was evaluated using rat tail-cut and liver punch biopsy models.

## 2. Materials and Methods

### 2.1. Materials

κ-carrageenan, glycerol, potassium dihydrogen phosphate, and dipotassium phosphate were purchased from Sigma-Aldrich (St. Louis, MO, USA). *C. officinalis* flower water-glycerol extract was purchased from Avena Lab-Farmadria d.o.o. (Vrsac, Serbia). Meadow polyfloral honey was purchased from a local organic food store (Belgrade, Serbia). Distilled water was used in all experiments.

### 2.2. Preparation of the Films

**Scheme 1:**
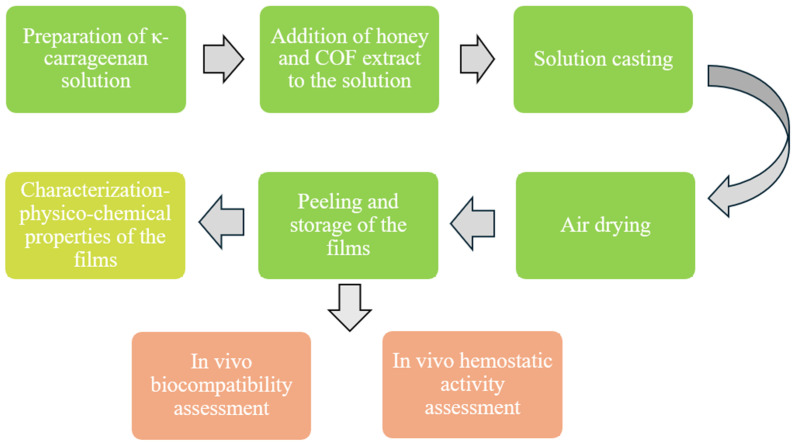
Schematic of the fabrication and characterization of the films.

The films were prepared using the solution casting method, starting from an aqueous solution of κ-carrageenan (1 wt%). κ-carrageenan was dissolved in distilled water and continuously stirred (600 rpm) at 60 °C for half an hour. Glycerol (5% *v*/*v*) was then added to the solution. Afterwards, different concentrations of honey and *C. officinalis* flower extract (COF) were carefully added to the solution and stirred (600 rpm) for 10 min. The solution was cast in a Petri dish and allowed to dry at room temperature to form a film. The dried films were peeled off and stored in Petri dishes in a desiccator prior to further testing. A diagram of the preparation and further investigation of the films is presented in Scheme 1. The composition and labeling of the prepared films are presented in Table 1.

### 2.3. Characterization of the Films

#### 2.3.1. Fourier Transform Infrared Spectroscopy (FTIR)

Structural characterization of the films was obtained by Fourier Transform Infrared Spectroscopy (FTIR)—ATR mode using Nicolet 6700 FTIR spectrometer (Thermo Fisher Scientific, Waltham, MA, USA), in 4000–600 cm^−1^ domain.

#### 2.3.2. Thickness, Weight Variation and Folding Endurance

The thickness of the films was measured at five random locations using a micrometer screw gauge. For weight variation, five different samples (1 × 1 cm^2^) were obtained from each film formulation and weighed. The results are displayed as the mean ± standard deviation (SD). The folding endurance of the films was evaluated as follows. The film (1 × 2 cm^2^) was repeatedly folded at an angle of 180 °at the same place until it started to break [48]. The adhesiveness of the films was visually monitored on human skin at several hand positions.

#### 2.3.3. Gel Fraction Measurement

The gel fraction was determined on film squares of 1 × 1 cm^2^. The films were dried for 24 h at 50 °C and then immersed in 15 mL of distilled water at 37 °C for 24 h. The films were removed from the water and dried at 50 °C until no weight changes was observed. The gel fraction (%) of the films was calculated using the following equation:

(1) $$Gel\;fraction\;\left(\%\right)=\frac{W_{d}}{W_{0}}$$

where *W*_0_ represents the initial weight of the film, and *W_d_* represents the weight of the film dried at 50 °C after immersion in distilled water. All experiments were performed in triplicate.

#### 2.3.4. Swelling Study

The swelling of the films was evaluated using a gravimetric method. Initially, dried films (1 × 1 cm^2^) were weighed and then immersed in phosphate buffer pH 7.4 (PBS) and pseudo extracellular fluid (PECF) [49]. At predetermined time intervals, the swollen films were removed from the swelling medium and weighed until a constant weight was reached. The degree of swelling was calculated using the following equation [50]:

(2) $$Swelling\;degree=\frac{W_{s}-W_{d}}{W_{d}}$$

where *W_d_* and *W_s_* are the weights of the dried and swollen films. All experiments were performed in triplicate.

#### 2.3.5. Moisture Uptake

The films were placed in a desiccator with calcium chloride for 24 h. Then, the dried films were weighed and transferred to a chamber with a relative humidity of 76%, obtained using a saturated sodium chloride solution for 72 h. The films were weighed, and the moisture uptake (%) was calculated using the following equation:

(3) $$Moisture\;uptake\;\left(\%\right)=\frac{W_{f}-W_{i}}{W_{i}}\times 100$$

where *W_i_* is the initial weight of the dried film, and *W_f_* is the weight of the film after exposure to humid air. All experiments were performed in triplicate.

#### 2.3.6. Water Retention Capacity

The ability of the films to hold and retain water was investigated. A film, swollen in PBS, was placed in a chamber with 76% relative humidity at 37 °C and weighed until a constant weight was reached. The weight remaining (%) was calculated using the following equation [51]:

(4) $$WR(\%)=\frac{W_{f}}{W_{i}}\times 100$$

where *W_i_* is the weight of the swollen film, and *W_f_* is the final weight. All experiments were performed in triplicate.

#### 2.3.7. In Vivo Biocompatibility Assay

##### Animals

In this study, three-month-old male Wistar rats weighing 250–300 g, obtained from the Department of Laboratory and Experimental Care and Use of Animals Unit of the Institute of Medical Research, Military Medical Academy (Belgrade, Serbia), were used. The rats were individually housed in polypropylene cages under standard conditions with a 12-h light/dark photoperiod, with food and water provided ad libitum. The experiment was approved by the Ethical Committee of the Faculty of Veterinary Medicine, University of Belgrade, and the Ministry of Agriculture, Forestry and Water Management—Veterinary Administration (decision number 0028966552024).

##### Experimental Design

Sterilized C3 and C7 films were subcutaneously implanted in 20 male Wistar rats. The rats were placed under general anesthesia with intraperitoneal injections of 75 mg/kg ketamine hydrochloride (Ketamidor 10%, 100 mg/mL, RICHTER PHARMA AG, Wels, Austria) and 10 mg/kg xylazine (Xylased 2%, BIOVETA, Ivanovice na Hané, Czech Republic). Following trichotomy and disinfection with povidone-iodine, one or two small incisions were made at the level of the midline of the thoracic spinal column parallell to the spine to create pockets in the subcutis. The films were implanted in these pockets, and some were left empty as control sites. Post-procedure, the animals received intramuscular analgesia with 5 mg/kg Ketoprofen (Ketonal, á 100 mg/2 mL, SANDOZ, Basel, Switzerland). The experiment lasted for 21 days, with euthanasia at four time periods: 3, 7, 14- and 21-days post-implantation (dpi). Five animals were euthanized at each time point, and skin tissue samples containing C3, C7, and control sites were collected. Prior to euthanasia animals were placed under general anesthesia following the same protocol used during surgery, and the hemostatic properties of the films were tested using rat tail-cut and liver punch biopsy models established by Morgan et al. [52]. In brief in the tail-cut model, a 4 cm segment was removed from the tip, and the tail stump was placed on pre-weighted filter paper to collect the dripping blood. Samples C3 and C7 were applied to the tail stump under gentle pressure, while the control groups received no treatment. Blood loss and time to hemostasis were recorded during the procedure. In the liver punch biopsy model, a midline laparotomy was performed, and the left lateral lobe of the liver was exposed. A pre-weighted filter paper was placed underneath, and an 8 mm biopsy punch (Kruuse, Langeskov, Denmark) was used to create a full-thickness injury within 2 mm of the lobe’s edge. C3 and C7 films were applied to the injury site in the treated groups, while the control group received no treatment. Data on blood loss and time to hemostasis were recorded during the procedure.

##### Histological Analyses

Skin tissue samples from the subcutis containing implanted films and control sites were fixed and processed conventionally for histological examination. Paraffin-embedded tissue blocks were serially sectioned at 5 μm using a semi-automatic microtome (Slee CUT 5062, Mainz, Germany) and stained with Hematoxylin-Eosin (H/E), Toluidine blue (TB), and Masson−Goldner (MG) staining kits (Merck Millipore, Darmstadt, Germany). The sections were mounted with DPX (phthalate-free) mounting medium (Fisher Scientific, Loughborough, UK) and examined using a standard Olympus CX31 microscope (Olympus Corporation, Tokyo, Japan).

##### Statistical Analysis

Blood loss and time to hemostasis data were analyzed using GraphPad Prism 9 software (GraphPad, San Diego, CA, USA). The normality of the data distribution was assessed using the Shapiro-Wilk test. All data were compared using one-way ANOVA followed by Tukey’s post-hoc test, except for blood loss data from the liver punch biopsy model, which were analyzed using the Kruskal-Wallis test followed by Dunn’s post-hoc test. The level of significance was set at *p* < 0.05.

## 3. Results and Discussion

### 3.1. Structural Characterization of the Films

**Figure 1:**
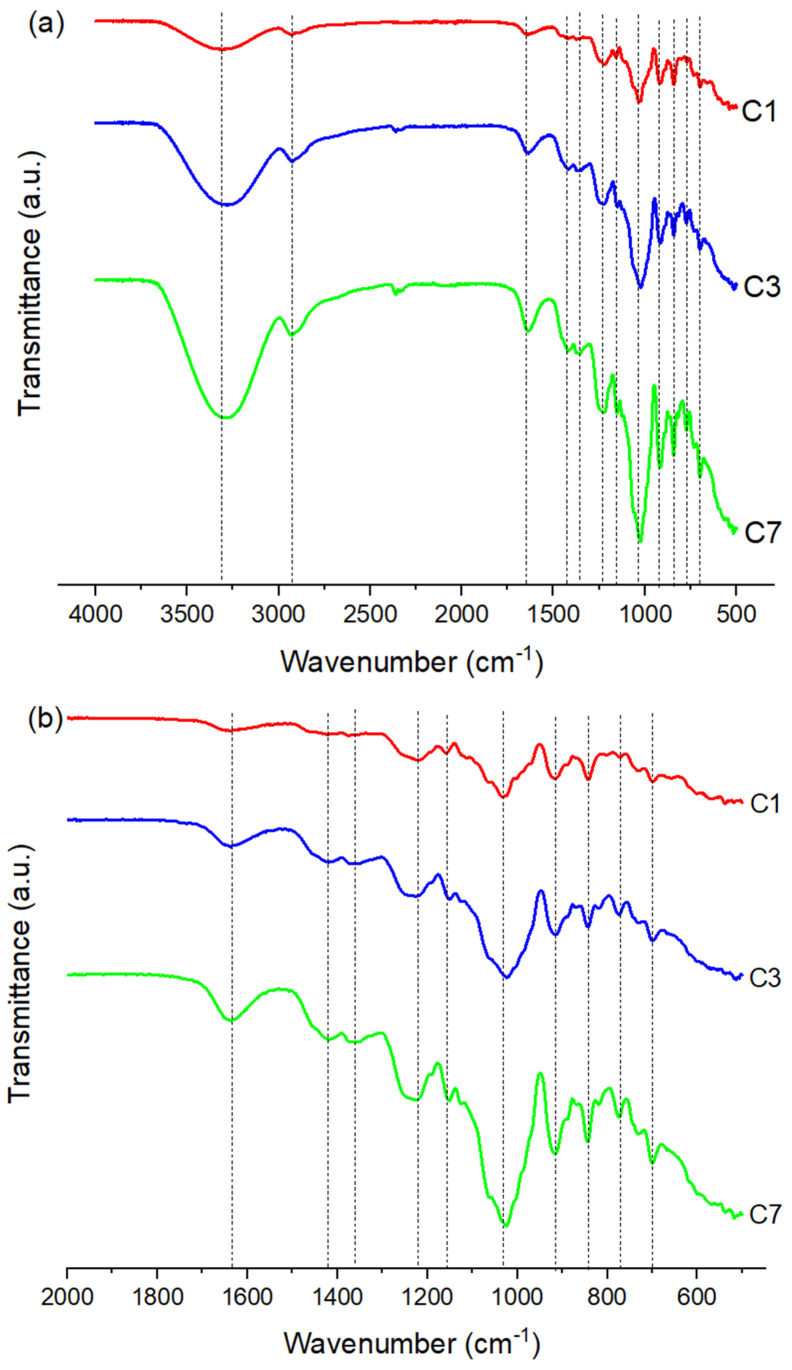
FTIR spectra of C1, C3, and C7 films in wavenumber range of (**a**) 4000–500 cm^−1^ and (**b**) 2000–500 cm^−1^.

Structural analysis was conducted on the unloaded C1 and C3 containing 5% honey and 5% COF extract and C7 loaded with 10% honey and 10% COF extract to confirm the successful fabrication of the films and examine the structural changes generated by the interactions between the bioactive compounds. The FTIR spectra are shown in Figure 1. The spectrum of C1 revealed peaks characteristic of κ-carrageenan at 3310 cm^−1^ assigned to -OH, 1230 cm^−1^ corresponding to sulfate ester groups, 842 cm^−1^ associated with S-O stretching, and 916 cm^−1^ originating from C-O vibration.

C3 and C7 films exhibited peaks characteristic of honey and COF extracts. The water content in honey and COF extract increased and shifted the intensity of the peak associated with the hydrophilic -OH group at 3310 cm^−1^ to 3294–3283 cm^−1^. The presence of honey is recognized by the peaks at 700, 775, 840, 916, 1025, 1160, 1360, 1430, 1640, and 2940 cm^−1^, which are mainly associated with carbohydrate molecules. Peaks at 1025 and 1160 cm^−1^ correspond to C-O and C-C stretching, while those at 1360 and 1430 cm^−1^ correspond to C-O-H and C-C-H bending [53,54]. The peaks characteristic of COF extract detected around 3290 cm^−1^ correspond to -OH stretching and H-bonding stretching from polyphenols; 2940 cm^−1^ is assigned to C-H symmetric and asymmetric stretching vibrations; 1360 cm^−1^ is related to C-O-H deformations in phenols and flavonoids, and 1220 cm^−1^ is associated with phenolic C-O stretching characteristic for flavonoids [55]. The absence of new peaks can be attributed to the lower concentrations of the honey and COF extracts. However, the slight shifts observed in the detected peaks indicate electrostatic interactions between the characteristic groups of κ-carrageenan, honey, and COF. These observations suggest the presence of the components and their interactions, confirming the successful preparation of the films.

### 3.2. Thickness, Weight Variation and Folding Endurance

**Figure 2:**
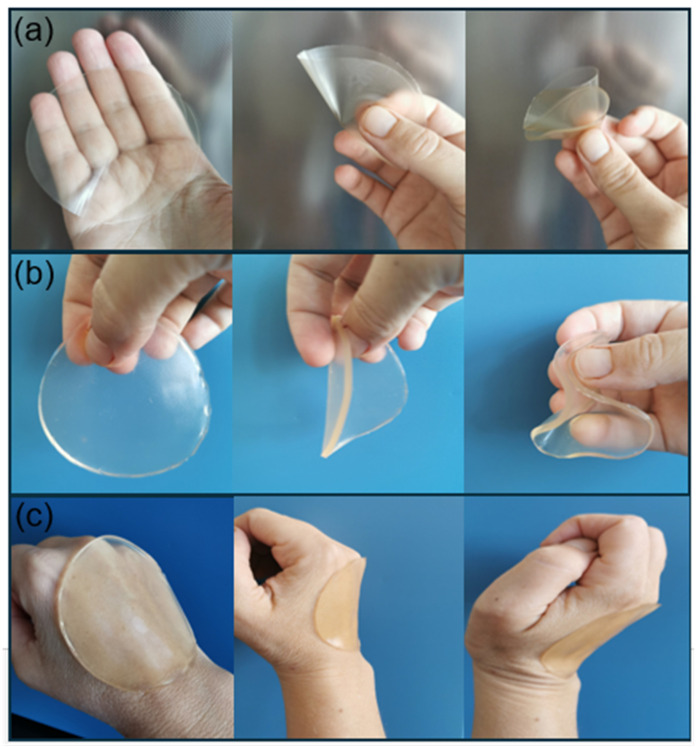
Folding ability of (**a**) dry, (**b**) swollen films, and (**c**) skin adhesivity.

The consistency in the thickness and weight of the films indicates the suitability of the method for producing a uniform biomaterial, as well as the possibility of scaling up. Folding endurance represents a measure of the film’s durability and resistance to deformation upon folding or bending, particularly when applied to areas of the body prone to movement. Values of the parameters are displayed in Table 2. The thickness, weight, and folding endurance of the films were measured in the range of 21.23 ± 3.41–25.22 ± 4.73 μm, 0.009 ± 0.002–0.015 ± 0.001 g, and 498 ± 5–508 ± 5 folds, where the increase in values was observed as honey and COF extract were incorporated and their concentrations were increased. The observed folding endurance indicates good mechanical strength, elasticity, and durability of the films (Figure 2a,b) [56]. The transparency of the fabricated films enables visual monitoring of the healing process, and their adhesiveness to human skin indicates proper attachment to the wound, easy application/removal, and protection of the wound (Figure 2c).

### 3.3. Gel Fraction

The gel fraction is a useful parameter for the formation of a polymeric network in the films, which indicates the insoluble portion of the material exposed to water or other solvents. It provides insights into the stability of the formed polymeric structure and the mechanical, swelling, and thermal properties of the fabricated films. Typically, higher values of gel fraction indicate more stable material with higher resistance to dissolution and degradation. However, when the film is assembled from a biopolymer, in this case polysaccharide, and non-polymeric bioactive compounds, the interpretation of the gel fraction may vary from this postulate due to the characteristic influence of honey and COF extract.

**Figure 3:**
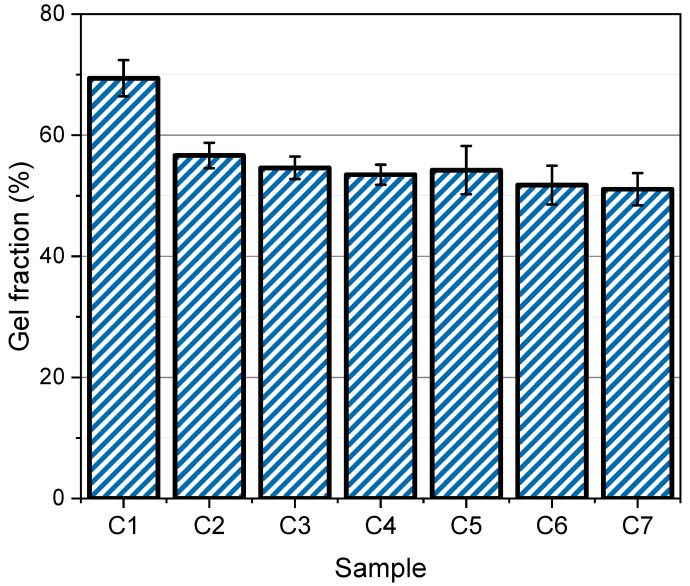
Gel fraction of the films.

The values obtained for the gel fractions of the fabricated films are displayed in Figure 3. The highest gel fraction was observed for the native κ-carrageenan film C1 (69.41%), while the lowest was exhibited by C7 film with 10% honey and 10% COF extract (51.08%). Although the introduction of additional components into the polymeric structure may be considered to strengthen and densify the material by filling the vacant space between polymeric chains (Figure 2), the presence of honey and COF extract lowered the gel fraction values. The addition of 5% honey (C2) induced a decrease in gel fraction (56.65%) compared to C1, while the doubled concentration of honey in C5 decreased it even more (54.24%). The presence of honey in the films can have an influence on stability and structural integrity of the polymeric network. Honey, which consists of water, sugars, and bioactive compounds such as polyphenols, can interfere with the gelation process by increasing the solubility of κ-carrageenan and reducing the crosslinking density of κ-carrageenan-based films. During the gelation process, honey compounds can compete with the reactive functional groups of κ-carrageenan, resulting in a decrease in the gel fraction [57]. The increase in the concentration of honey from 5% to 10% followed this narrative, where the values of the gel fraction slightly decreased. The introduction of the COF extract as an additional bioactive compound influenced the gel fraction in the same manner. The films containing the COF extract, C3 and C6, exhibited lower gel fractions (54.60% and 51.76%) than C2 and C5. Furthermore, increasing COF extract concentration to 10% in C4 and C7 decreased the obtained values (53.48% and 51.07%). This trend is consistent with the observations on honey’s influence on κ-carrageenan gelation process, because this plant extract consists of various bioactive compounds, such as polyphenols, steroids, terpenoids, carotenoids, triterpenoids, and flavonoids. Although some of these molecules can act as crosslinking agents and increase the gel fraction, they can also disrupt the formation of the polymeric network by increasing the dissolution of the polymer with additional water and hindering the gelation process [58].

### 3.4. Swelling of the Films

The most characteristic property of polymer-based biomaterials is their swelling ability. The ability to controllably absorb significant amounts of fluid renders them one of the most versatile biomaterials. Accordingly, they have been explored for various biomedical applications, such as controlled delivery systems, scaffolds, and dressings. Polymer-based film wound dressings represent an advanced approach for wound treatment, with significantly improved healing efficiency compared to traditional methods, owing to swelling. Specifically tailored films can accelerate the healing process by providing optimal moisture to the wound bed, absorbing excess excreted exudates, and delivering bioactive molecules to actively promote the regeneration of damaged skin tissue [59].

**Figure 4:**
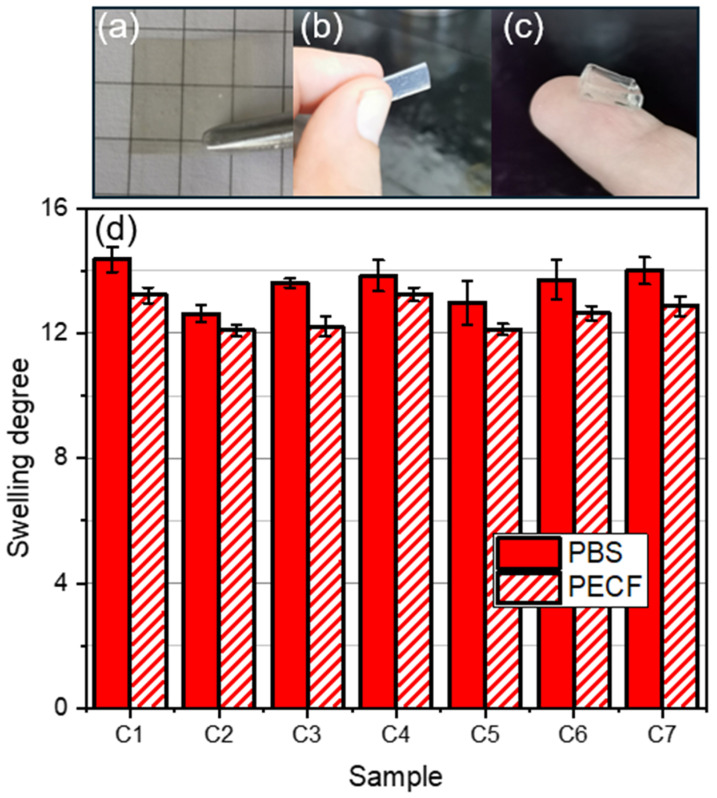
Swelling of the films: (**a**) dry sample, (**b**) sample during the swelling process, (**c**) swollen sample, and (**d**) degree of swelling.

The swelling capacity of the fabricated films was influenced by the presence and increased concentration of honey and COF extract, as well as the type of swelling medium (Figure 4d). The highest swelling degree values were observed for the native κ-carrageenan film (C1) in both swelling media, 14.37 (PBS) and 13.22 (PECF). All films exhibited lower swelling in PECF than in to PBS, due to the higher concentration of ionic species that interact with the ionized functional groups of κ-carrageenan, honey, and COF extract. The films with honey and COF extract had lower *q_e_* values, where the effect of each bioactive compound was observed. The introduction of 5% honey in C2 induced a decrease in *q_e_* to 12.63 (PBS) and 12.09 (PECF) compared to C1. The increase in honey content (10%) in C5 enhanced swelling to 12.99 (PBS) and 12.12 (PECF), but did not exceed the capacity of C1. At a concentration of 5%, honey can disrupt the polymeric network to some extent and interfere with the hydrophilic sites on κ-carrageenan polymer chains. Swelling was present but decreased compared to the native κ-carrageenan polymeric network, where the water molecules were absorbed unhindered. At 10%, although the free space between the polymer chains is more occupied, the influence of the specific nature of honey prevails, and the amount of absorbed water is greater than that at lower concentrations. Therefore, the swelling of the films was influenced by the concentration of honey and the chemical composition of the films. Salva et al. [60] showed that with an increase in honey concentration from 1 to 6%, the swelling capacity of chitosan/hyaluronic acid hydrogels decreased. Conversely, higher concentrations of Manuka honey (10%, 20%, and 30%) were found to enhance the swelling capacity of 2-hydroxyethyl methacrylate/gelatin hydrogels [61]. The effect of the COF extract on the swelling capacity of the hydrogel films was noticeable through a slight increase in *q_e_*. The physical and chemical interactions between molecules from honey, extract, and κ-carrageenan can affect the structure, lead to the formation of additional water-bonding sites, and alter the specific surface area [62]. The results revealed that the addition of COF extract increased swelling, and the increase in its concentration from 5 to 10% emphasized this effect.

According to the results, the films based on κ-carrageenan loaded with honey and COF extract, prepared using the described procedure, possess the capacity to absorb significant amounts of water, indicating their potential for application in the treatment of wounds with higher quantities of exudates. By absorbing excess liquid from wounds, optimal moisture can be maintained.

### 3.5. Moisture Uptake

Moisture uptake is an important feature of films and has an influence their performance as wound dressing materials. The ability to absorb moisture is related to the flexibility of the films and their adaptation to variations in physiological and environmental factors. Moisture uptake influences the barrier properties of film dressings and the preservation of optimal moisture at the wound site.

**Figure 5:**
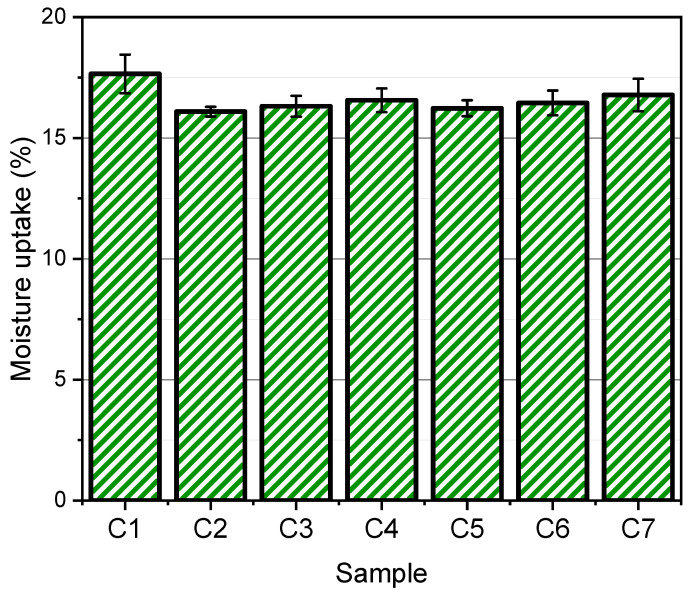
Moisture uptake of the films.

The values of moisture uptake obtained after exposing the dried films to humid air (76%) are displayed in Figure 5. The conducted study confirmed the influence of chemical composition on the sorption properties of the samples. The values of moisture uptake were in the range of 16.09–17.65%, where the highest and lowest values were exhibited by C1 and the lowest by C2. Since the moisture uptake represents the ability to absorb moisture from the air, a parallel with the swelling ability of the films can be drawn. The sorption of moisture from air followed the same pattern as the sorption of the swelling medium, except that the differences between the samples were more subtle. The introduction of honey (5%) in C2 decreased moisture uptake compared to C1, while the increase in honey concentration (10%) in C5 slightly improved this feature (16.23%). The presence of an additional hydrophilic component, the COF extract, enhanced moisture uptake, with C3 exhibited 16.31% and C6 16.45%. The increase in the COF extract concentration to 10% increased the values; thus, C4 exhibited 16.56% and C7 16.78% of moisture uptake. Even though hydrophilic compounds were introduced into the polymeric structure, the native κ-carrageenan film showed the highest sorption of moisture. As observed in the previous section, the ability to absorb water molecules inside the polymeric matrix highly depends on the physical and chemical interactions of the components. Native κ-carrageenan forms a uniform and stable polymeric network, where hydrophilic groups attract water molecules undisturbed by the interactions that occur when honey and COF extract are involved. The influence of honey and COF extract on the ability to absorb moisture absorption suggests that the specific surface and surface hydrophilicity can be altered by subtle variations in the chemical composition of the films.

### 3.6. Water Retention

**Figure 6:**
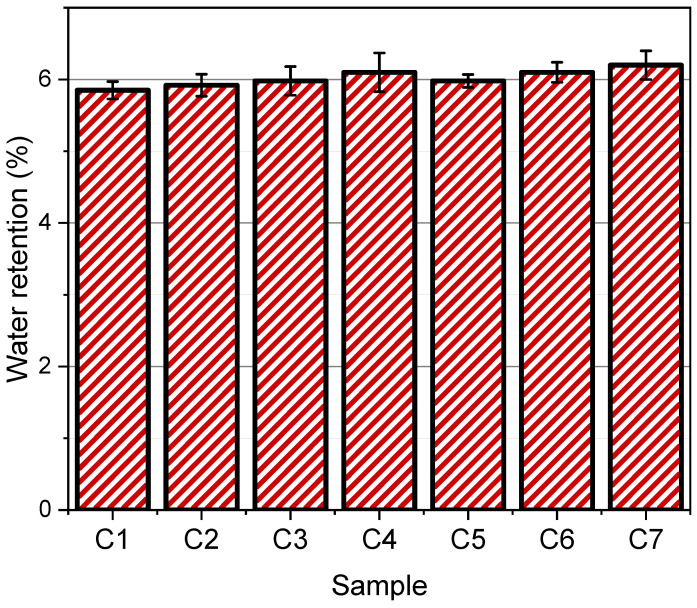
Water retention of the films.

While efficiently absorbing excess exudate from the wound, a film dressing should provide optimal moisture by retaining a certain amount of water inside the polymeric matrix. The water retention capacity is closely related to the chemical composition of the films, which influences the swelling capacity and moisture uptake, all three of which are known as essential indicators of efficient management of fluids excreted from the wound. The water retention of the fabricated films was evaluated, and the results are presented in Figure 6. Under relatively humid conditions, a swollen film begins to dry when no additional fluids, such as saline, are available to maintain the moisture of the dressing. The loss of water is affected by the chemical composition of the films and the ability of the fabricated structure to hold the absorbed water. Although the native κ-carrageenan sample C1 exhibited the highest degree of swelling, the water retention was the lowest (5.85%). The introduction of honey (5%) in C2, C3, and C4, and the increase in its concentration to 10% in C5, C6, and C7 increased the water retention capacity compared to that of C1. The addition of COF extract at 5% (C3 and C4) and 10% (C6 and C7) further enhanced this property, which is consistent with their swelling capacity. Therefore, the highest water retention (6.20%) was exhibited by C7. As discussed earlier, the polymeric network of native κ-carrageenan film possesses the greatest capacity to absorb water molecules due to the abundance of vacant space between the macromolecular chains and unhindered interaction between hydrophilic groups. However, the capacity to hold absorbed water was lower than that of the honey- and COF-loaded hydrogel films. This observation indicates stronger interactions between bioactive components and water molecules, which contributes to improved moisture retention. In summary, the results obtained indicate that the fabricated films can provide additional moisture to the wound bed by holding about 6% of the water excreted from the wound inside the polymeric matrix.

### 3.7. In Vivo Biocompatibility Assessment

**Figure 7:**
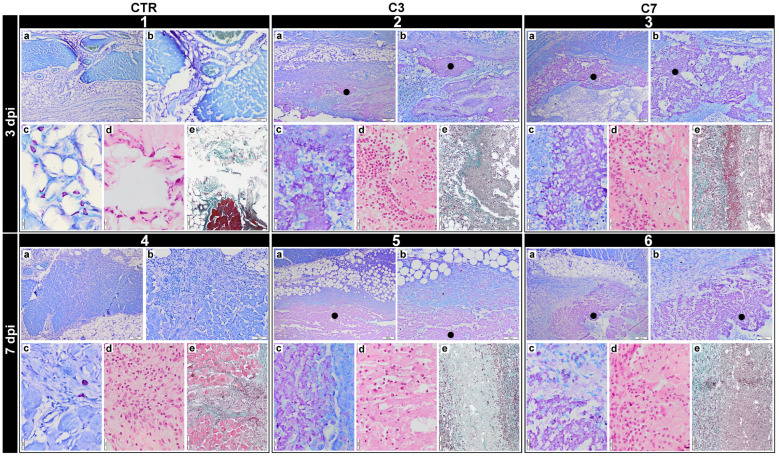
In vivo biocompatibility assessment of the films at three- and seven-days post-implantation (dpi): representative photomicrographs of skin sections from: (**1**) control (CTR) animals (3 dpi); (**2**) animals implanted with C3 (3 dpi); (**3**) animals implanted with C7 (3 dpi); (**4**) CTR animals (7 dpi); (**5**) animals implanted with C3 (7 dpi); and (**6**) animals implanted with C7 (7 dpi). Toluidine blue staining is shown in all images labeled (**a**–**c**); hematoxylin and eosin staining in all images labeled (**d**); and Masson−Goldner staining in all images labeled (**e**). Skin sections were viewed using a scanning (4×) objective (scale bar: 200 µm) in all images labeled (**a**); a low-power (10×) objective (scale bar: 100 µm) in all images labeled (**b**,**e**); and a high-power (40×) objective (scale bar: 20 µm) in all images labeled (**c**,**d**). The black dot (•) indicates the location of the implanted films.

The host response is the first and one of the most significant factors in determining the successful future use of novel biomaterials. Although in vitro studies can provide some insights, implantation remains the best practice for understanding this response [63]. Implantation of synthetic materials is characterized by a classical foreign body response, with a capsule of collagen fibers oriented parallel to the surface of the biomaterial and a scarcity of blood [63,64]. The host response to natural biomaterials, such as those used in our study, differs significantly and is represented by the innate immune response and remodeling phenotype of macrophages [63]. C3 and C7 samples were chosen for in vivo evaluation as samples loaded with both honey and COF extract in lower (5%) and higher (10%) concentrations, to investigate their influence and reveal the optimal composition of the fabricated films. Representative photomicrographs of skin samples at 3- and 7-dpi are shown in Figure 7. At 3-dpi pronounced vascularization, mast cell, and granulocyte infiltration were evident at the control sites (Figure 7(1a–1e)). In the treated groups, C3 and C7 films were clearly visible in the subcutis due to their purple staining in the TB-stained sections (Figure 7(2a–2c,3a–3c)). Higher magnification revealed cellular infiltration around the samples and initial degradation, with granulocytes being the predominant cell type (Figure 7(2c,2d,3c,3d)). Although a fully formed capsule was absent in the treated groups, the organization of collagen fibers with a fascicle pattern on the borders of the implant was evident (Figure 7(2e,3e)). The deposition of collagen on the borders of the film was more prominent in C3, while vascularization of the border region was greater in the C7 group (Figure 7(2e,3e)). The control cites at 7-dpi were similar to the prior period, with only a difference in the decrease in infiltrating granulocytes (Figure 7(4a–4e)). Sections from the treated groups showed slightly more pronounced degradation of the films, the infiltrate was less dense, and although granulocytes were still present, they were no longer dominant (Figure 7(5a–5d,6a–6d)). Macrophages were also evident in the infiltrate at this time period (Figure 7(5c,5d,6c,6d)). The collagen fibers were organized in the same manner as at 3-dpi, but were less densely packed (Figure 7(5e,6e)).

**Figure 8:**
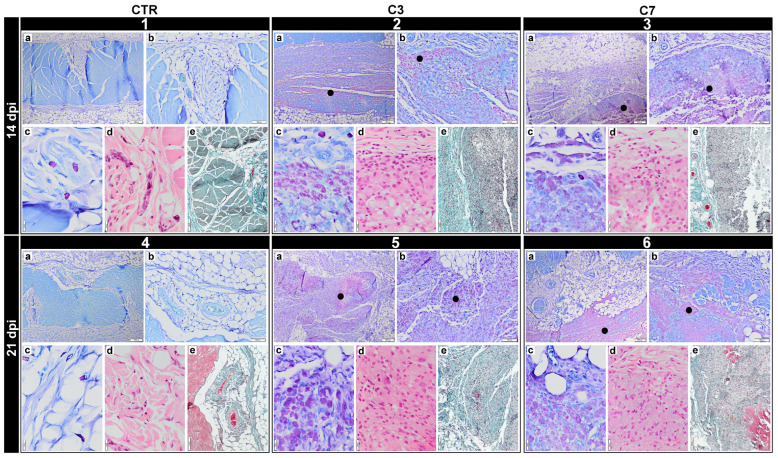
In vivo biocompatibility assessment of the films at 14- and 21-days post-implantation (dpi): representative photomicrographs of skin sections from (**1**) control (CTR) animals (14 dpi), (**2**) animals implanted with C3 (14 dpi), (**3**) animals implanted with C7 (14 dpi), (**4**) CTR animals (21 dpi), (**5**) animals implanted with C3 (21 dpi), and (**6**) animals implanted with C7 (21 dpi). Toluidine blue staining is shown in all images labeled (**a**–**c**); hematoxylin and eosin staining in all images labeled (**d**); and Masson−Goldner staining in all images labeled (**e**). Skin sections were viewed using a scanning (4×) objective (scale bar: 200 µm) in all images labeled (**a**); a low-power (10×) objective (scale bar: 100 µm) in all images labeled (**b**,**e**); and a high-power (40×) objective (scale bar: 20 µm) in all images labeled (**c**,**d**). The black dot (•) indicates the location of the implanted film.

Figure 8 shows representative photomicrographs of the skin samples at 14 and 21 dpi. Vascularization and mast cell infiltration were still evident in the control sites at 14 dpi, but granulocytes were absent (Figure 8(1a–1e)). Examination of the subcutis region in the treated groups revealed further degradation of the films (Figure 8(2a–2d,3a–3d)). Macrophages were the predominant cell type in the infiltrate (Figure 8(2c,2d,3c,3d)), and their phagocytic activity was evident in TB-stained sections, with purple granules clearly visible in the cytoplasm (Figure 8(2d,3d)). The area surrounding the implanted film showed collagen deposition with a mixed fiber orientation and pronounced vascularization, especially in the C7 group (Figure 8(2a–2c,2e,3a–3d)). The control sites at 21-dpi were consistent with those in the previous period (Figure 8(4a–4e)). In the treated groups, the films were mostly phagocytosed, with the purple stain in the TB-stained sections reflecting numerous granules in macrophages (Figure 8(5a–5d,6a–6d)). The deposition and organization of collagen fibers and vascularization were the same as at 14-dpi (Figure 8(5e,6e)).

The absence of a capsule around the implanted films and the transition of collagen fiber organization from parallel to mixed-orientation indicate the remodeling of the extracellular matrix and are in accordance with the host response to natural biomaterials described in the literature [63]. The infiltrate was first characterized by the dominant presence of granulocytes, which were substituted with macrophages in later periods; however, the appearance of giant cells formed by the fusion of macrophages was not detected. Similar results were described by Popa et al. [65] regarding carrageenan-based hydrogel biocompatibility. In contrast to Popa et al. [65], who observed complete hydrogel absorption by 15-dpi, our study observed hydrogel persistence at later time points, likely due to differences in formulations. Giusto et al. [66] showed that a pectin hydrogel containing honey also revealed no typical foreign body response and demonstrated neovascularization around the implant. Most studies assessing the biocompatibility of biomaterials with *Calendula officinalis* are still being conducted in vitro. Rad et al. [67] proposed 10% *Calendula officinalis* extract as the optimal concentration for scaffold fabrication based on cytotoxicity examination, as it was the highest concentration that did not show a significant decrease in cell viability compared to lower concentrations (1%, 2.5%, and 5%). *Calendula officinalis* extracts also promoted angiogenesis in both chorioallantois membrane models and acute wound healing studies [68]. The region bordering the implanted films showed good vascularization in both treatment groups, which may be attributed to the angiogenic effects of honey and *Calendula officinalis* extract, with enhanced vascularization in the C7 group, possibly due to the higher concentrations of these compounds in the film formulation. The observed vascularization, collagen deposition, and alignment of collagen fibers can be attributed to the effects of polyphenols and sugars from honey, as well as triterpenoids, flavonoids, and carotenoids present in the *Calendula officinalis* flower extract. The histological findings suggest that the biodegradation of the evaluated films did not induce inflammation and that the degradation byproducts were biologically safe within the tested period.

### 3.8. Assessment of Hemostatic Properties

**Figure 9:**
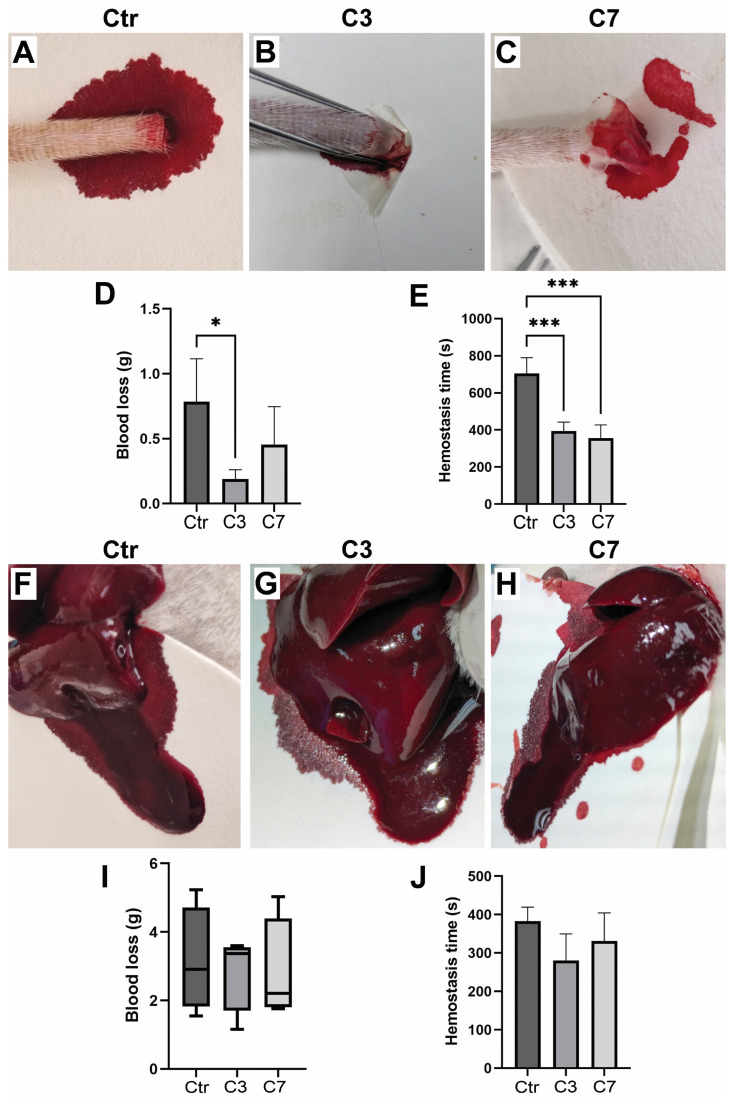
The assessment of hemostatic properties: photographs of hemorrhages in the rat tail-cut model taken at the time of hemostasis the (**A**) control, (**B**) C3, and (**C**) C7 groups, accompanied by graphs presenting (**D**) blood loss and (**E**) time to hemostasis (data presented as mean ± standard deviation). Photographs of hemorrhages in the rat liver punch biopsy model taken when the bleeding stopped: (**F**) control, (**G**) C3, and (**H**) C7 groups along with graphs showing (**I**) blood loss and (**J**) time to hemostasis (data presented as mean ± standard deviation). (* and *** indicate *p* < 0.05 and *p* < 0.001, respectively).

Due to the variable efficiency of traditional hemostatic materials in various clinical scenarios, there is a growing interest in developing novel polymer-based materials, particularly those synthesized from natural polymers, as evident from preclinical research trends [69]. Carrageenan and *Calendula officinalis* are promising compounds for hemostasis research [23,47,70]. Madruga et al. [70] showed that carboxymethyl-kappa-carrageenan nanofibers enhanced thrombocyte adhesion, activation, and clotting, potentially explained by the preferential adsorption of fibrinogen over albumin on the nanofibers. *Calendula officinalis* flowers and leaves are also known for their ability to enhance hemostasis [46,71]. Ayyanahalli Matta et al. [47] showed that *Calendula officinalis* oil significantly reduced clot formation time compared to the control. Conversely, examining the effects of natural honey on human platelets revealed inhibitory effects on both platelet aggregation and whole blood coagulation [32]. Our examination of hemostatic properties using the rat tail-cut model demonstrated that C3 and C7 films reduced blood loss (0.1875 ± 0.0732 g, 0.4550 ± 0.2922 g) compared to the control (0.7837 ± 0.3319 g), with significance observed only in the C3 group (*p* < 0.05) (Figure 9A–D). The lesser reduction in blood loss observed in the C7 group could be explained by the inhibitory properties of honey, considering its higher concentration in C7. Both C3 and C7 achieved hemostasis significantly faster (393.75 ± 47.97 s, 355.75 ± 71.42 s) than the control group (704.25 ± 85.29 s) (*p* < 0.001) (Figure 9E). In the rat liver punch biopsy model, both treated groups exhibited less blood loss (C3 2.8725 ± 1.1476 g, C7 2.8025 ± 1.5174 g) and shorter times to hemostasis (C3 303.25 ± 77.90 s, C7 331.00 ± 73.42 s) than the control group (3.1475 ± 1.54.13 g, 383.00 ± 36.53 s) (Figure 9F–J), although the differences were not significant. The absence of significance could be explained by the presence of different types of capillaries in the liver and tail, which are sinusoidal and continuous types, respectively. The differences in capillary types and endothelial glycocalyx [72] present in the two models may be the reason for the different efficacies of the films perceived in our study, warranting further investigation at the molecular level.

## 4. Conclusions

This paper details the synthesis and characterization of novel κ-carrageenan-based films loaded with meadow poly floral honey and *Calendula officinalis* flower extract as novel natural wound healing therapy. The synergistic activity of the biologically potent components was thoroughly evaluated using various characterization methods appropriate for the potential application of the fabricated biomaterials. Light, transparent, skin-adhesive, and durable films exhibited appropriate sorption capacity and barrier properties. Subcutaneously implanted samples induced vascularization, deposition of collagen and orientation of collagen fibers. During in vivo biocompatibility assessment, the films were completely phagocytosed, followed by gradual biodegradation. The rat tail-cut and liver punch biopsy models revealed hemostatic capacity through reduced blood loss and hemostasis time compared to the control. Future work will focus on the optimization of chemical composition of the films and evaluating their antimicrobial potential for use in the treatment of infected wounds, all of which will precede clinical trials.

## Figures and Tables

**Table 1:** Composition of the films.

Formulation	Honey (wt% of Polymer Content)	COF (wt% of Polymer Content)	Glycerol (wt% of Polymer Content)
C1	-	-	5
C2	5	-	5
C3	5	5	5
C4	5	10	5
C5	10	-	5
C6	10	5	5
C7	10	10	5

**Table 2:** Thickness, weight variation, and folding endurance of the films.

Formulation	Thickness (μm) ± SD	Weight Variation (g) ± SD	Folding Endurance (Number of Folds) ± SD
C1	21.23 ± 3.41	0.009 ± 0.001	498 ± 5
C2	22.05 ± 4.28	0.010 ± 0.002	501 ± 3
C3	22.98 ± 3.77	0.011 ± 0.001	500 ± 4
C4	23.56 ± 3.82	0.011 ± 0.003	502 ± 5
C5	24.15 ± 4.09	0.012 ± 0.001	503 ± 3
C6	24.84 ± 5.01	0.014 ± 0.002	507 ± 5
C7	25.22 ± 4.73	0.015 ± 0.001	508 ± 4

## Data Availability

The data supporting the findings of this study are available from the corresponding author upon reasonable request. The data are not publicly available due to ongoing research.
